# Targeted Delivery of *Toxoplasma gondii* Antigens to Dendritic Cells Promote Immunogenicity and Protective Efficiency against Toxoplasmosis

**DOI:** 10.3389/fimmu.2018.00317

**Published:** 2018-02-20

**Authors:** Zineb Lakhrif, Alexis Moreau, Bruno Hérault, Anne Di-Tommaso, Matthieu Juste, Nathalie Moiré, Isabelle Dimier-Poisson, Marie-Noëlle Mévélec, Nicolas Aubrey

**Affiliations:** ^1^ISP, INRA, Université Tours, Nouzilly, France

**Keywords:** single-chain fragment variable fragment antibody, DEC-205, SAG1, vaccination, toxoplasmosis

## Abstract

Toxoplasmosis is a major public health problem and the development of a human vaccine is of high priority. Efficient vaccination against *Toxoplasma gondii* requires both a mucosal and systemic Th1 immune response. Moreover, dendritic cells play a critical role in orchestrating the innate immune functions and driving specific adaptive immunity to *T. gondii*. In this study, we explore an original vaccination strategy that combines administration *via* mucosal and systemic routes of fusion proteins able to target the major *T. gondii* surface antigen SAG1 to DCs using an antibody fragment single-chain fragment variable (scFv) directed against DEC205 endocytic receptor. Our results show that SAG1 targeting to DCs by scFv *via* intranasal and subcutaneous administration improved protection against chronic *T. gondii* infection. A marked reduction in brain parasite burden is observed when compared with the intranasal or the subcutaneous route alone. DC targeting improved both local and systemic humoral and cellular immune responses and potentiated more specifically the Th1 response profile by more efficient production of IFN-γ, interleukin-2, IgG2a, and nasal IgA. This study provides evidence of the potential of DC targeting for the development of new vaccines against a range of *Apicomplexa* parasites.

## Introduction

Toxoplasmosis is a worldwide foodborne zoonosis caused by the intracellular protozoan parasite *Toxoplasma gondii*. In animals, it causes considerable economic losses in livestock (1), and infected meat constitutes a parasitic source for human infection (2). Infection commonly starts at the intestinal mucosal surface, spreads into the body, and is rapidly confined to some host tissues (heart, brain, eyes, muscles) throughout host life. Usually asymptomatic in hosts, toxoplasmosis can be a serious threat to public health, and it may lead to severe or lethal damage when associated with immunosuppressive states or when transmitted to the fetus during pregnancy (3). Acquired resistance to *T. gondii* infection is mediated by a mucosal and systemic Th1 cellular immunity (4), which depends mainly on the ability of T cells to produce IFN-γ (5). Dendritic cells play a key role in cellular immunity through interleukin (IL)-12 secretion, the major cytokine triggering adaptive immune response by promoting IFN-γ production (6). Vaccines that are able to enhance potent and broad mucosal and systemic Th1 T cell responses can therefore provide protective immunity to *Toxoplasma* infection. Attempts to develop subunit vaccines against *T. gondii* have focused mainly on SAG1, the major surface protein of tachyzoites. SAG1, the best-characterized antigen, is composed of two domains, D1 and D2. The D2 domain links to the glycosylphosphatidylinositol anchor, whereas the D1 domain is exposed outwardly (7, 8). SAG1 is the first protein involved in the invasion process (9) and is highly conserved in *T. gondii* strains (10). SAG1 contains T and B neutralizing epitopes (7, 11, 12), and subunit SAG1 vaccines have been shown to induce both antigen-specific humoral and T cell responses and to confer protection against acute (13), chronic (14, 15), and congenital toxoplasmosis (16, 17). However, these protections are partial, and new strategies to improve the efficacy of subunit SAG1 vaccines are necessary.

The crucial role of DCs in the initiation and regulation of adaptive immunity has led to their use in dendritic cell-based vaccination (18). It has been documented that following loading with pathogenic antigens and adoptive transfer, DCs mediate protection against a wide spectrum of infectious diseases, including toxoplasmosis. We previously showed that DCs pulsed with *T. gondii* antigen elicit protective immunity against chronic toxoplasmosis in mice (19, 20). However, it is not feasible to use *ex vivo* antigen-loaded DCs for first-line prophylactic vaccination. Targeting dendritic cells *in situ* through antigen-DC receptors will circumvent this problem. Indeed, this new strategy is effective against viral (21, 22), bacterial (23), and parasitic infections (24) and can be explained by the facility of exposing antigens to dendritic cells and their regulated presentation pathways. The outcome of these studies emphasizes that targeted delivery of antigen to DC surface endocytosis receptors such as C-type lectin receptor (CLR) increases antibody and cell-mediated responses (25). Myeloid cells, including dendritic cells and macrophages, express a large number of C-type lectins (26). DEC205 has been extensively employed for targeted delivery of antigens to DCs in murine and human studies (18). DEC205 is a member of the MMR family of type I transmembrane CLRs. In mice, DEC205 is expressed on cortical thymic epithelium, thymic medullary DCs (CD11c+, CD8α+), and subsets of peripheral DCs (splenic, lymph node DCs, dermal, interstitial DCs, and Langerhans cells) (27). Targeting antigen to the DEC205 receptor improved humoral and cellular immune responses when DC-activating agents or adjuvants such as polyinosinique–polycytidylique acid (Poly I:C) were also administered (21, 24, 28).

Most studies used whole monoclonal antibodies to target antigens to dendritic cells. Single-chain fragment variable (scFv) antibodies are less frequently used in targeted vaccination strategies, and the few existing studies are based on a gene vaccination approach (29, 30). The only protein vaccine approach, based on the fusion protein scFv-antigen, is used by Coconi-Linares et al (22) to target EDIII of envelope dengue virus to DEC205. The use of scFv, rather than complete antibodies, offers several advantages for antigen targeting. Their smaller size increases the bioavailability in tissue (31). More importantly, scFv lack an Fc domain that reduces the deleterious immunogenicity in host cells. In particular, they cannot bind to other cells *via* Fc receptors, which may reduce unspecific uptake, improving DEC205-specific antigen delivery (29). Furthermore, scFv production is less expensive than that of whole antibodies (32).

A key consideration to produce a successful vaccine is the choice of appropriate vaccination routes. The combination of two routes eliciting both systemic and mucosal immune responses is important for protection against *T. gondii* (14, 15, 33). Indeed, mucosal immunity is the first line of defense and systemic immunity provides protection against parasite dissemination. DC targeting *via* the intranasal (i.n.) route was investigated to improve mucosal vaccine efficiency (23). Furthermore, the interest in combined immunization routes in the efficient induction of immunity has been previously confirmed in different models using intradermal and sublingual vaccinations (34) and by i.n. and intramuscular (35) or i.n. and intradermal vaccination (36).

Interestingly, it has been shown in mice that, among other Toll-like receptor (TLR) agonists, Poly (I:C) is the most effective inducer of the CD4+ Th1 response profile in the DEC205 targeting strategy. Airways present a particular histological landscape of several cell types, including epithelial cells, macrophages, and different subsets of dendritic cells, which can respond in different way to TLR activation. Errea et al. (37) showed that triggering TLR3 by i.n. administration elicits a strong local response compared to TLR4 or TLR5. More specifically, i.n. administration of Poly (I:C) has been previously used in a DEC205 targeting strategy (23).

By using this background information, we constructed a fusion protein to target the major surface antigen SAG1 to dendritic cells using an antibody fragment scFv directed against DEC205 endocytic receptors to improve the protective immunity against *T. gondii*. The D1 domain of the SAG1 protein contains both B and T cell epitopes. It was important to evaluate the immunogenic potential of D1 targeting. Thus, the vaccination strategy was based on targeting of D1 and D1D2 domains of SAG1 with the fusion proteins SA1 and SA2, respectively. The produced proteins were characterized structurally and functionally. The studies performed aimed to determine vaccination effector pathways, SA1 and SA2 protein vaccine efficiency, and the effectiveness of targeting against untargeted antigen. The humoral and cellular immune responses induced by targeted and untargeted proteins were analyzed. Our findings clearly indicate that SAG1 targeting to DCs through i.n. and subcutaneous (s.c.) routes is a promising approach to improve protection against chronic toxoplasmosis.

## Materials and Methods

### Engineering and Production of the Targeted or the Untargeted Proteins

#### Targeted Parasitic Antigens

SAG1 antigen was selected for this study. The coding sequence for the D1 domain (48–181 amino acids) or D1D2 domains (48–302 amino acids) of SAG1 was amplified from pcDNA3-SAG1 (38) by PCR using primers *D1 Rev* (AGCTAGCCCTCTTGTTGCCAA), *D1b For* (TTCTCGAGTTAGTGATGGTGATGGTGATGTGAGGCTCTGGCTTGTACT), and *D2b For* (TTCTCGAGTTAGTGATGGTGATGGTGATGGGCAAACTCCAGTTTCA CGGTACAGTGATG) (Eurogentec), which introduce *NheI* and *XhoI* restriction sites as well as a histidine-encoding sequence.

#### pMT Anti-DEC205 scFv Plasmid Generation

Single-chain fragment variable anti-DEC205 (DC15) result from the association of the heavy and light variable domains, as published by Demangel et al. (29), *via* a (Gly_4_Ser)_3_ peptide link and from a spacer GGGAS and peptide histidine flag in the *C*-terminal. The synthetic gene (GeneArt) was inserted in the plasmid pMT/BiP/V5 His A (pMT) (Invitrogen). The generated plasmid pMT-DC15 was further modified to construct a universal plasmid with three unique sites: *PstI, NheI*, and *XhoI*, allowing insertion of other scFv between these restriction sites. Complementary primers containing the desired mutation were used according to the QuikChange II Site-Directed Mutagenesis Kit protocol.

#### Fusion of D1 or D1D2 Antigens to the Anti-DEC205 scFv

The pMT-DC15-D1 (pMT-SA1) and pMT-DC15-D1D2 (pMT-SA2) were obtained by the insertion of D1 or D1D2 sequences between the *NheI/XhoI* restriction sites, respectively.

#### Untargeted Antigens Cloning

For the untargeted control, D1D2 was amplified with the primers D1RS Rev (AGATCTCCCCCTCTTGTTGCCAATCAAGTTG) and HisX For (TTCTCGAGTTAGTGATGGTG) (Eurogentec) that allows the use of *BglII* and *XhoI* restriction enzymes to insert its sequence in pMT. Complementary primers D1RSSD Rev (GCCTTTGTTGGCCTCTCGCTCGGGTCGGATCCCCCTCTTGTTGCCAATCAAG) and D1RSSD For (CTTGATTGGCAACAAGAGGGGGATCCGACCCGAGCGAGAGGCCAA CAAAGG) were used to mutate the RS in SD. This provided the pMT-SAG1t, keeping the same initial sequence relative to the target sequence of D1D2.

The generated plasmids were amplified using DH5α cells and purified using the Endofree Plasmid Giga Kit (Qiagen) according to the manufacturer’s instructions.

### Expression and Purification of Fusion Proteins

The proteins were produced in the *Drosophila* Schneider 2 cell line as previously described (39). The proteins were purified by affinity chromatography with a His resin (Miltenyi). The purified proteins SAG1t (D1D2), SA1, and SA2 were resolved by size-exclusion chromatography (FPLC) on a Superdex 75 10/300 GL column (molecular mass range 3,000–70,000) (GE Healthcare Life Sciences) with an Akta purifier. The column was loaded with 0.5 nmol of each protein.

Protein concentrations were determined with a UV detector at 280 nm. The characteristics of each protein, molecular mass, and molar extinction coefficient were defined using http://web.expasy.org/protparam/: SAG1t (261 amino acids, 27,596.2 Da, ε = 0.787/mg mL cm), SA1 (383 amino acids, 40,007.7 Da, ε = 1.767/mg mL cm), and SA2 (504 amino acids, 53,711.1 Da, ε = 1.514/mg mL cm).

### Immunoblot Analysis

Each protein was dissolved in 12% SDS-PAGE gel under non-reducing or reducing conditions and subsequently transferred to nitrocellulose membranes (GE Healthcare). Membranes were blocked with 5% non-fat milk diluted in TNT (15 mM Tris–HCl, 140 mM NaCl, 0.05% Tween 20) for 1 h at room temperature and then incubated overnight at 4°C with rabbit anti-His polyclonal antibody (1:1,000, Sigma) or pooled sera from serum from an infected mouse, diluted at 1:100. After washes with TNT, bound antibodies were detected using anti-mouse IgG or anti-rabbit IgG alkaline phosphatase conjugate (1:5,000, Sigma). Alkaline phosphatase activity was detected using BCIP/NBT substrat (Promega). ProSieve QuadColor Protein Markers (Lonza) were used.

### Binding Assay

Binding assays were performed using bone marrow dendritic cells (BMDCs) and splenic cells expressing the mouse DEC205.

Bone marrow dendritic cells were collected from femurs and tibiae of CBA/J mice as described previously in Ref. (33). BMDCs were plated at 3 × 10^6^ cells/mL and cultured at 37°C, 5% CO_2_ in 10 mL complete medium [RPMI-1640 medium containing 10% heat-inactivated fetal bovine serum (FBS), 10 mM HEPES, 1 mM pyruvate, 2 mM l-glutamine, 100 U/mL penicillin, streptomycin (All from PAN BioTech), 1% non-essential amino acids (GIBCO), and 50 µM 2β-mercaptoethanol], and 20 ng/mL of GMCSF from J558 cells line supernatant. Media was replaced every 2 days, and cells were used at day 10 in which DEC205 expression reach a maximum.

Single-cell splenocyte suspensions were obtained from spleen first pressed and then filtered through a nylon mesh. Hypotonic shock (0.155 M NH_4_Cl, pH 7.4) was used to remove splenic erythrocytes. The cells were then suspended in RPMI-1640 medium supplemented with 5% FBS, 25 mM HEPES, 2 mM l-glutamine, 1 mM sodium pyruvate, 50 µM 2β-mercaptoethanol, and 1 mM penicillin–streptomycin.

Bone marrow dendritic cells and splenic cells were incubated overnight at 4°C with 11 µM of each protein (targeted or untargeted) and then stained with anti-His FITC antibody (Miltenyi) for 30 min at 4°C. 10,000 events were acquired for the analysis of binding. Samples were acquired using BD FACSCalibur cytometer (BD Biosciences) and analyzed using the CellQuest software (BD Biosciences).

### Immunofluorescence Assay

5 × 10^5^ BMDCs were plated and cultured for 24 h on coverslips. Cells were incubated overnight at 4°C with 6 µM of targeted or untargeted proteins and fixed in 4% paraformaldehyde, blocked with phosphate-buffered saline (PBS)-SVF 5%. After incubation with anti-His FITC antibody, protein binding was detected by immunofluorescence microscopy (Zeiss Germany).

### Activation and Maturation of BMDCs

On day 10 of BMDCs culture, cells were incubated with 3.25 µM of SA2 (10 µg/mL), 3.25 µM of SAG1t (5 µg/mL), or medium for 24 h at 37°C, 5% CO_2_. The next day, supernatants were collected to assay cytokines (IL-12p40, IL-6, TNF-α, IL-1β, IL-10, and IL-13) or chemokines (CCL3, CCL5, CCL20, and MCP-1). Cytokine and chemokine concentrations were determined using commercial ELISA kits according to the manufacturer’s instructions (eBioscience and RD system, respectively).

Cells were stained with FITC-conjugated antibodies reacting with mouse CD40 (clone 3/23), CD80 (clone 16-10A1), CD86 (clone B7-2 BL1), and I-A/I-E (2G9) molecules. All monoclonal antibodies were purchased from BD Biosciences. 5,000 events were acquired for the analysis of binding. Samples were acquired using BD FACSCalibur cytometer (BD Biosciences) and analyzed using the CellQuest software (BD Biosciences).

### Mice Immunization and Challenge

The 6-week-old female CBA/J (H-2^k^) mice (Janvier, Le Genest St. Isle, France) resistant to acute toxoplasmosis infection and susceptible to cysts formation in chronic infection were used in this study. Groups of 8 or 12 mice were immunized s.c., i.n. or using combined routes three times at 2-week intervals with the different protein vaccines formulated with 50 µg of Poly I:C for the s.c. route and 10 µg for the i.n. route. The equimolar quantities of each injected protein are presented in Table 1.

**Table 1:** Summary of performed treatments.

	Subcutaneous		Intranasal	
SA1		12.87 µg		6.62 µg
D1D2		8.66 µg		4.46 µg
	1.8 µm		0.9 µM	
SA2		16.86 µg		8.67 µg

Two weeks after the last immunization, mice (8/group) were orally challenged with 15 cysts of the 76K *T. gondii* strain. Protection was evaluated 1 month after challenge by analyzing the cyst load in brain tissue. Mouse brains were homogenized in 5 mL of RPMI medium, and the number of tissue cysts per brain was determined microscopically by counting 10 samples (10 µL each) of each homogenate.

### Humoral Response following Immunization

Titers of SAG1-specific IgG antibodies and IgG subclasses were performed by ELISA on sera collected 2 weeks after the third immunization. Flat-bottomed 96-well plates (Nunc) were coated overnight with 4 µg/mL SAG1t, SA1, and P30 protein (45–198 amino acids, Prospec, Israel) in 50 mM carbonate buffer (pH 9.6). The plates were washed with PBS–Tween 0.05% and blocked with PBS–4% bovine serum albumin (BSA) (Amresco). Serial dilutions of serum were performed in PBS–BSA 4%, and the plates were incubated for 2 h at 37°C. The plates were then washed again and incubated for 1 h at 37°C with Goat anti-Mouse IgG alkaline phosphatase (1:5,000, Sigma), rat anti-mouse IgG1 alkaline phosphatase (X56), or rat anti-mouse IgG2a alkaline phosphatase (R19-15) (both at 1:1,000, BD Pharmingen). After washes, para-nitro-phenyl-phosphate (Amresco) diluted in DEA-HCl at 10 mg/mL was added. The optical density of each sample was read at 405 nm. Results are expressed in log2 titers.

Nasal washes were collected 1 week after the last immunization by repeated flushing and aspiration of 1 mL of PBS containing 1 mM phenylmethylsulfonyl fluoride (Sigma). Intestinal washes were performed with syringe by passing 5 mL of PBS 1 mM phenylmethylsulfonyl fluoride through the gut. Nasal and intestinal IgA were detected by Western blotting. SAG1t protein was separated on a polyacrylamide gel at 12% and transferred onto a nitrocellulose membrane. The membrane was blocked in PBS–BSA 4%. Nasal and intestinal washes were then added and incubated overnight at 4°C. After washes in TNT, IgA-bound antibodies were detected using goat anti-mouse IgA alkaline phosphatase conjugate (1:1,000, Sigma) for 1 h. An alkaline phosphatase substrate NBT-BCIP (Promega) was diluted according to manufacturer’s instructions and used to detect the antibodies.

### Immunofluorescence Assay of *T. gondii* Tachyzoites with Sera of Immunized Mice

Slides coated with acetone-treated tachyzoites of the RH strain were incubated with sera of immunized mice diluted at 1:10 in PBS for 30 min at 37°C and then washed three times with PBS. The tachyzoites were incubated with anti-mouse IgG-TRITC secondary antibody (1:200, Sigma) for 30 min at 37°C and washed three times with PBS. The slides were then mounted and observed by immunofluorescence microscopy (Zeiss Germany). A pooled serum from infected and uninfected mice was used as positive and negative controls, respectively.

### *T. gondii* Extract (TE)

Tachyzoites of the RH strain were obtained by serial passaging in human foreskin fibroblast (HFF) cell monolayers and used to prepare TE containing both cytoplasmic and membrane antigens. Tachyzoites in PBS were sonicated and centrifuged as previously described (40). The protein concentration was determined by the Micro BCA protein assay reagent kit using BSA as the standard (Pierce, Rockford, Ill.). TE was stored in aliquots at −20°C until use.

### Cellular Response following Immunization

Four mice per group were sacrificed 7 days after the last immunization. Cell suspensions from spleens and mesenteric lymph nodes (MLNs) were prepared as described above. Cells (5 × 10^5^ cells/well) were stimulated in triplicate with 10 µg/mL TE. Concanavalin A (1 mg/mL) was used as a positive control for proliferation. Supernatants were harvested and assayed for IL-2 after 24 h and for IFN-γ, IL-5, IL-10, and IL-13 activity after 72 h (ELISA kits, eBioscience).

To analyze whether CD4+ and/or CD8+ T cells contribute to the protective immune response, CD4+ and CD8+ T cells from control and immunized mice were purified using CD4 (L3T4) and CD8α (Ly-2) MicroBeads (Miltenyi). As described above, BMDCs were loaded for 24 h with SAG1t and SA2, and these differently stimulated DCs were subsequently used to stimulate the purified CD4+ and CD8+ T cells at 1:2 ratio (5 × 10^5^ BMDCs and 1 × 10^6^ T cells). After 72 h, supernatants were harvested, and cytokines (IFN-γ and IL-2) were assayed using eBiosciences ELISA kit.

Further, involvement of CD4+ or CD8+ T cells in the secretion of cytokines was studied in whole splenocyte populations from immunized mice. Spleen cell suspensions were stimulated in triplicate with 10 µg/mL of TE, 0.15 µM of SAG1t (4 µg/mL), or SA2 (8 µg/mL) and in the presence or absence of 20 µg/mL of anti-mouse CD4 (clone GK1.5) or anti-mouse CD8α (clone 53-6.72) functional antibodies from eBioscience. Supernatants were harvested after 72 h and assayed for IFN-γ activity (ELISA kits, eBioscience).

### Statistical Analyses

Statistical significance was analyzed using GraphPad Prism software. Statistical analysis was done by one-way ANOVA followed by a Tukey’s multiple comparison test and Student’s *t*-test. For variables that were not normally distributed, statistical analysis was performed using a Kruskal–Wallis test followed by Dunn’s multiple comparison test, and the results were expressed as median and interquartile range. *P* < 0.05 was considered to be statistically significant.

## Results

### Structural Characterization of the Produced Proteins

**Figure 1:**
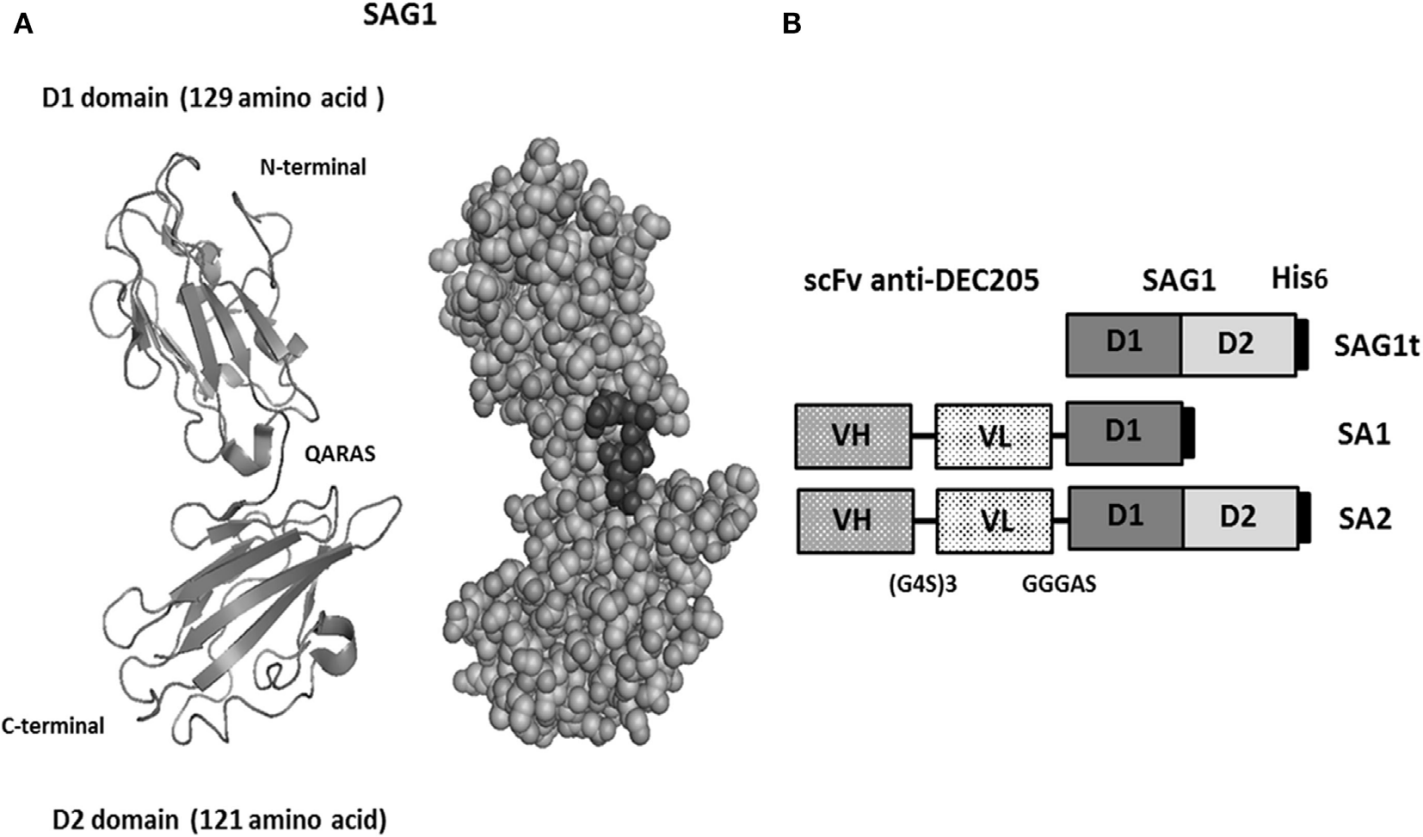
Design of vaccine proteins. **(A)** Structural 1YNT pdb model of SAG1 protein with two domains (D1 in *N*-terminal and D2 in *C*-terminal). **(B)** Schematic representation of the untargeted (SAG1t) and the targeted antigens (SA1 and SA2).

**Figure 2:**
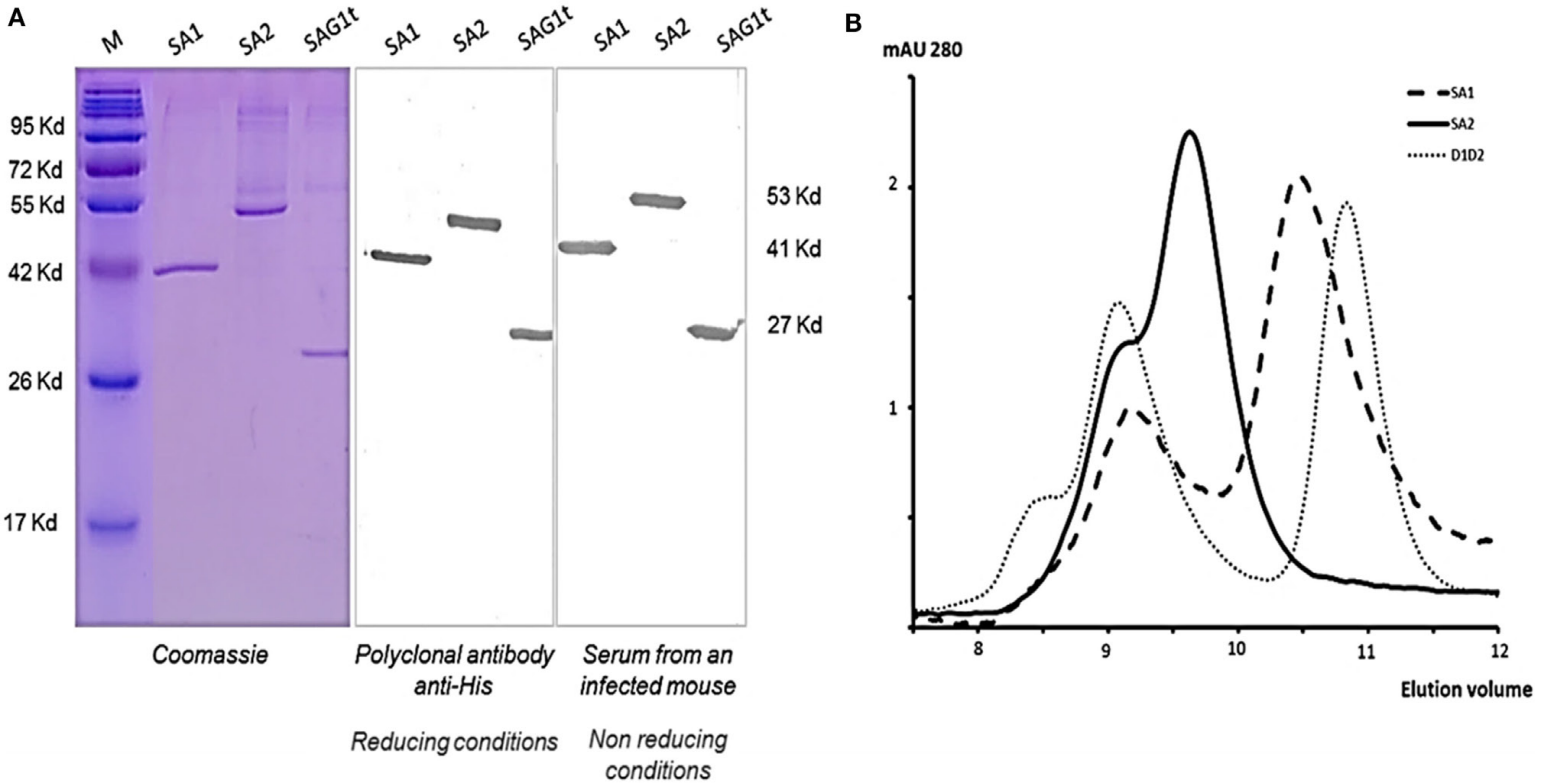
Structural characterization of the produced proteins. **(A)** Analysis of purified proteins from insect cells supernatant by Coomassie Blue staining and Western blot using rabbit polyclonal antibody anti-His or serum from an infected mouse under reducing and non-reducing conditions, respectively. **(B)** Representative elution profile of SAG1t, SA1, and SA2 size exclusion chromatography. 0.5 nmol of each purified protein was injected onto a Superdex 75 HR 10/30 column.

The major surface protein of *T. gondii* (Figure 1A) was targeted to dendritic cells using two fusion proteins SA1 and SA2, which target, respectively, D1 and D1D2 domains (Figure 1B). An untargeted control construct SAG1t was also designed (Figure 1B). The constructs carried a His tag in *C*-terminal for proteins purification and detection. Untargeted and targeted proteins were produced in S2 *Drosophila* cells, purified by nickel-chelator agarose affinity chromatography, and the yield of purified proteins was around 17 mg/L for SAG1t and SA2 but lower for SA1 (8 mg/L). In addition to the best yield, the SA2 fusion protein is more stable than SA1. The purified proteins showed electrophoretic behavior compatible with the expected molecular weights (27 kDa for SAG1t, 41 kDa for SA1, and 53 kDa for SA2) (Figure 2A). They are recognized not only by an anti-His polyclonal antibody but also by serum from an infected mouse, which confirms the antigenicity of these proteins. The size-exclusion chromatography showed a major peak corresponding to the monomeric form of SAG1t, SA1, and SA2 proteins (Figure 2B). The three vaccine protein preparations are of equal quality.

### Functional Characterization of the scFv

**Figure 3:**
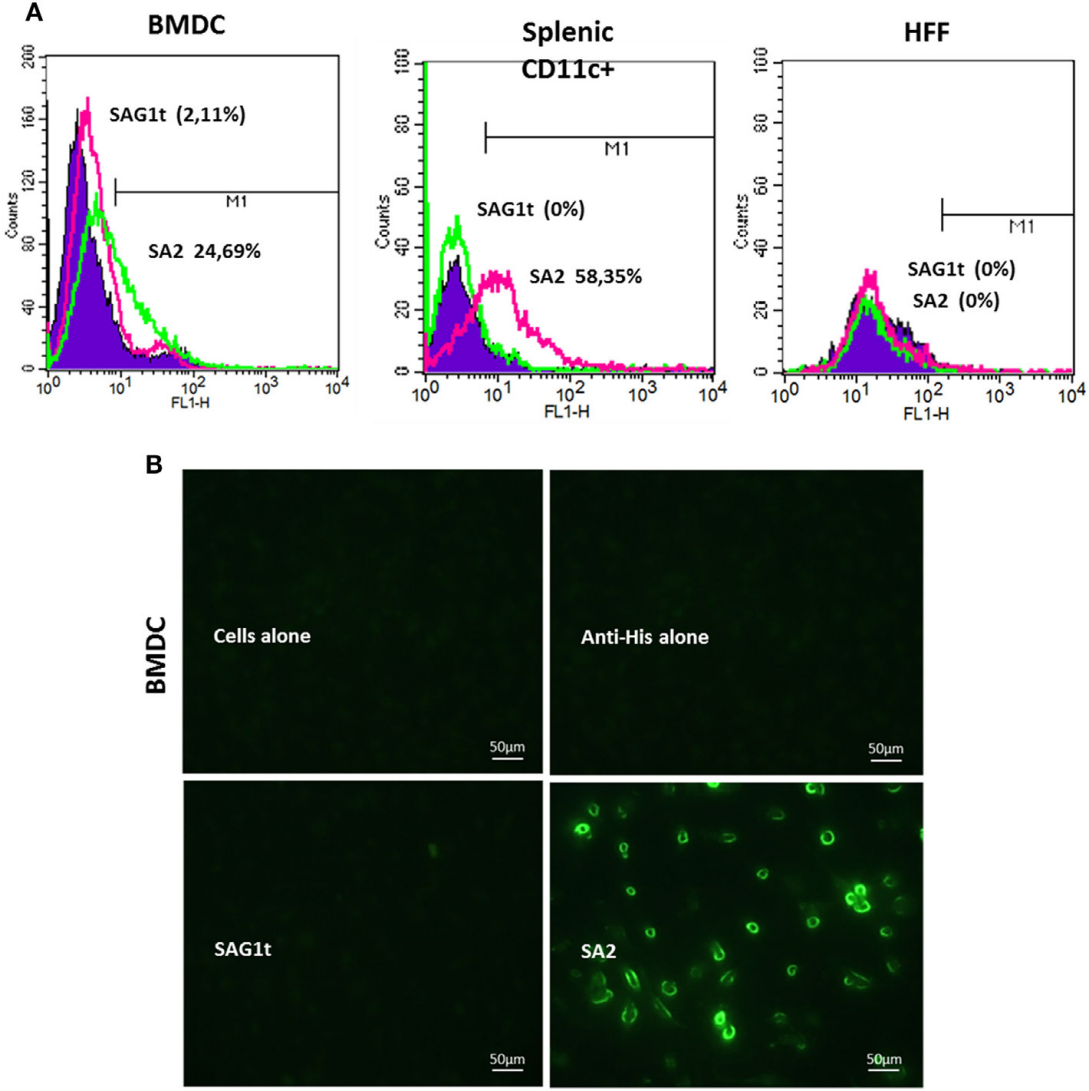
*In vitro* recognition and binding of the targeted protein to the cell surface. **(A)** Analysis by flow cytometry of the protein binding on bone marrow dendritic cells (BMDCs), CD11c+ splenic cells, and human foreskin fibroblast (HFF) cells. Cells were incubated overnight at 4°C with targeted or untargeted proteins, and the protein binding was assessed with anti-His FITC by flow cytometry. As a negative control, the cells were stained only with anti-His FITC. Binding was detected on CD11c+ gated cells (10,000 events). The percentages of cells labeled with anti-His FITC were calculated as change in fluorescence intensity compared to isotype control. **(B)** Immunofluorescence analysis of protein binding on BMDC cells. Coverslips were incubated overnight at 4°C with targeted or untargeted proteins, then fixed and blocked. Protein binding was revealed by immunofluorescence microscopy after incubation with anti-His FITC antibody.

We evaluated the ability of the scFv used in our constructs to bind to DEC205+ cells by flow cytometry. For this, the best targeted protein in terms of yield and stability (SA2) was used. As shown in Figure 3A, the SA2 protein stained 25% of BMDCs and 58% of splenic CD11c+ cells, while only 2% of BMDCs and splenic CD11c+ cells were labeled when incubated with SAG1t. These percentages are similar to those obtained with the anti-DEC205 monoclonal antibody incubated with the same cells (23% for BMDCs and 63% for splenic CD11c+, data not shown). As expected, SA2 did not bind to other cells such as HFF or HEK 293T (Human Embryonic Kidney), which do not express DEC205. These results were confirmed by immunofluorescence analysis (Figure 3B). SA2, but not SAG1t, labeled with anti-His FITC, was found to bind to BMDC cells.

### Targeting Promotes BMDC Maturation *In Vitro*

The activation of antigen-presenting cells as dendritic cells is one of the critical steps toward an effective immune response *in vivo*. Induced maturation and activation of BMDCs with targeted SAG1 was tested. For this purpose, BMDCs were incubated for 24 h with SA2 or SAG1t. ELISA was performed to measure the anti-/pro-inflammatory cytokines (IL-12p40, IL-6, IL-1β, and TNF-α), Th2 cytokines (IL-5 and IL-13), and the chemokines (CCL3, CCL5, CCL20, and MCP-1) production. The level of expression of co-stimulatory molecules (CD40, CD80, CD86, and MHCII) was measured by flow cytometry.

**Figure 4:**
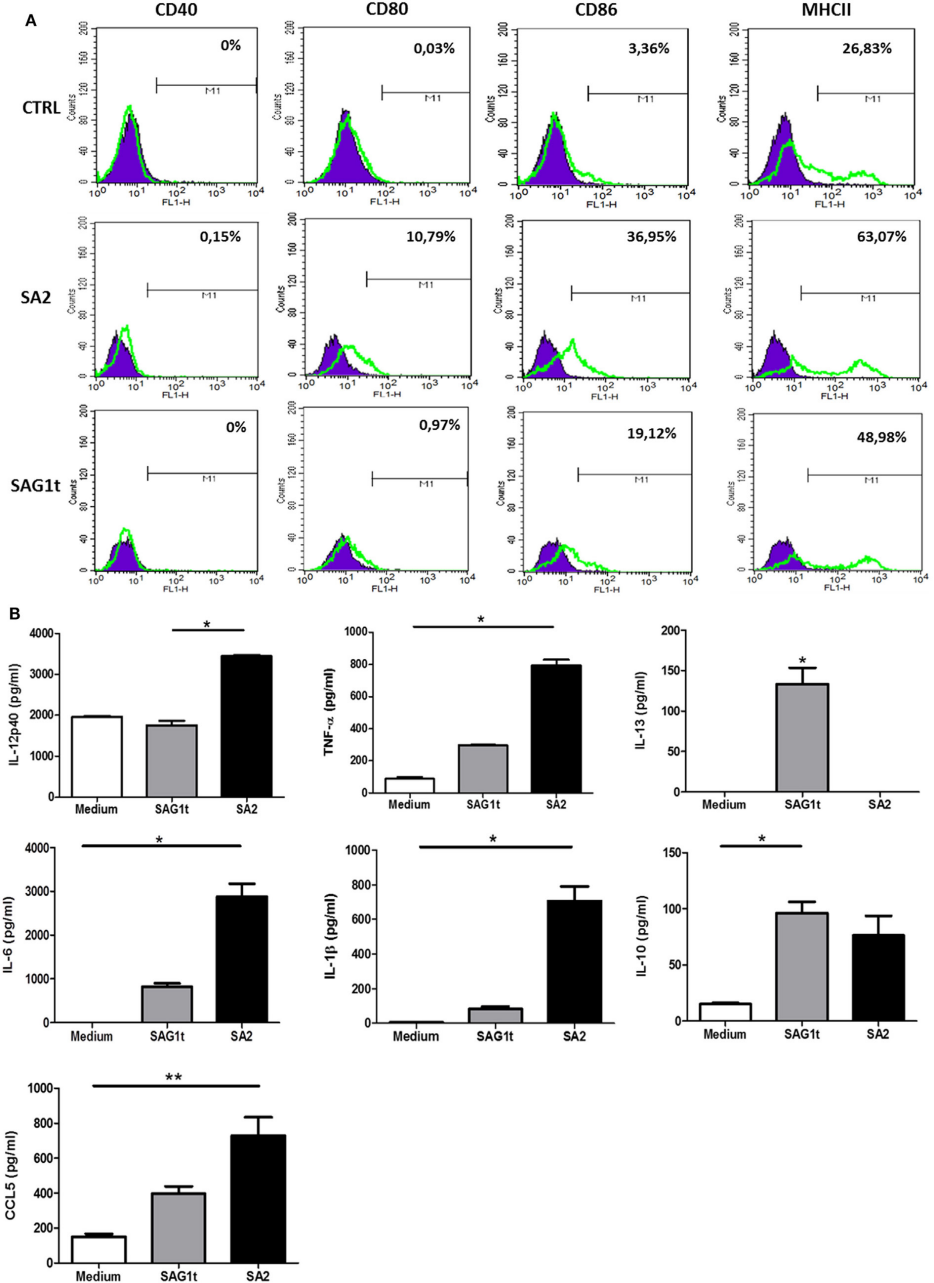
Maturation and activation of bone marrow dendritic cells (BMDCs) following incubation with targeted or untargeted SAG1 protein. **(A)** Analysis of surface expression of maturation markers. BMDCs at day 10 of culture were stimulated 24 h with SA2 or SAG1t or left unstimulated (medium). Surface maturation markers (CD40, CD80, CD86, and MHCII) were assessed by flow cytometry. **(B)** Cytokines and chemokines secretion. Cytokines and chemokines were assayed by ELISA in the supernatants of stimulated cells. Results are expressed as median ± interquartile and represent one of two independent experiments. *P < 0.05; **P < 0.01.

Upregulation of CD80, CD86, and MHCII was detected in BMDCs stimulated with SA2 compared with untreated BMDCs (Figure 4A). In parallel, the expression profile of cytokines and chemokines was evaluated. Figure 4B showed that BMDCs stimulated with SA2 produced significant levels of IL-12p40, TNF-α, IL-6, IL-1β cytokines, and CCL5 chemokine, whereas BMDCs stimulated with SAG1t did not produce significant amounts of these molecules compared with control. SAG1 maturation induced significant production of IL-13. No specific release of CCL3, CCL20, and MCP-1 chemokines was observed in any culture supernatant (data not shown). In conclusion, SA2 stimulation improves BMDC maturation and directs the immune response towards a Th1 profile.

### Major Effectors of Targeting Protective Efficiency

#### Simultaneous Mucosal and s.c. Vaccination Efficiently Protects Mice against Chronic Toxoplasmosis

**Figure 5:**
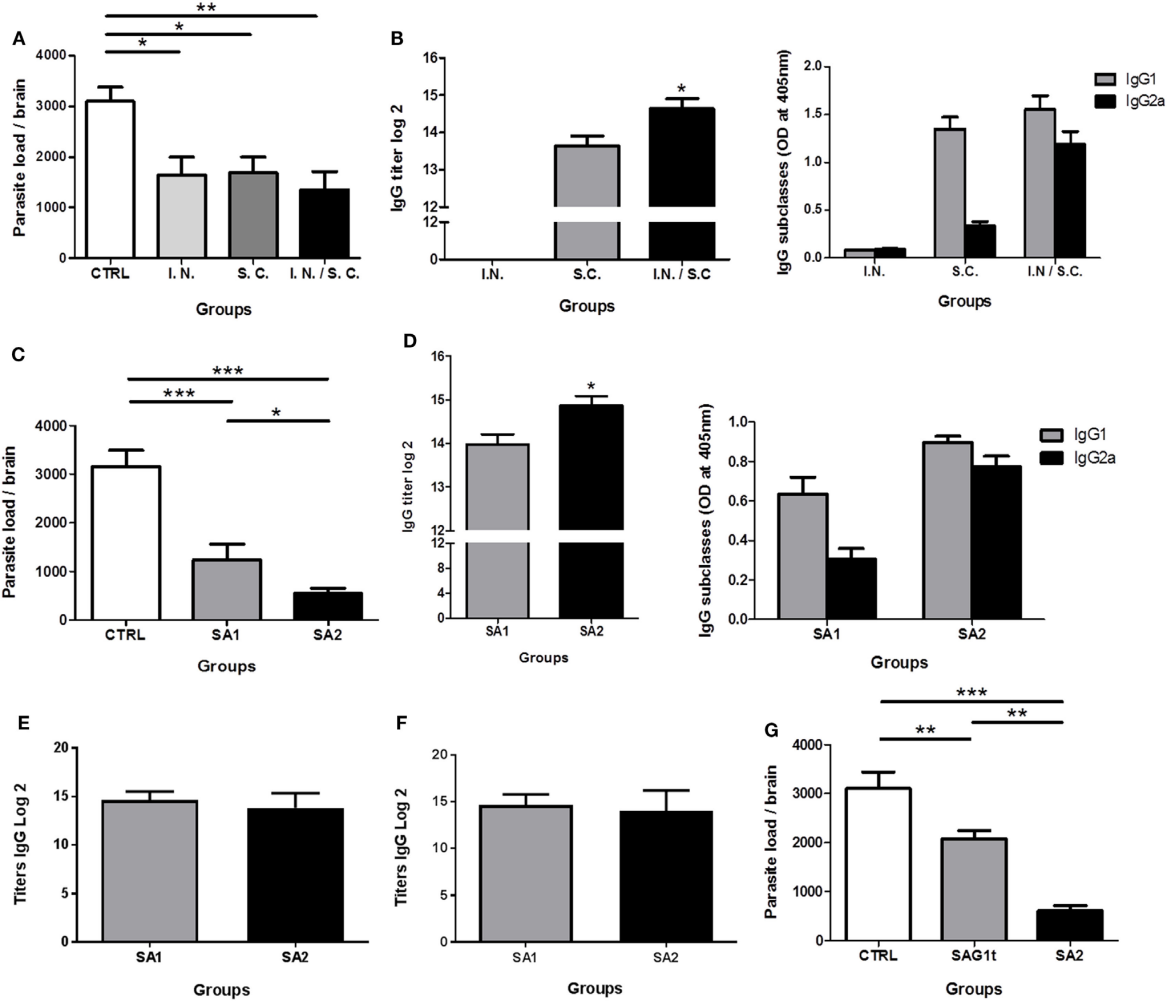
Evaluation of the protection against chronic toxoplasmosis and humoral response induced following immunization. CBA/J mice (8/group) were primed and boosted twice with SA1, SA2, or SAG1t formulated with polyinosinique-polycytidylique acid (Poly I:C) by different administration routes. Control mice received Poly I:C by the combined routes. **(A,B)** Mice immunization with SA1 by intranasal, subcutaneous, and combined routes. **(C,D)** Mice immunization with SA1 and SA2 by combined routes. **(D)** Mice immunization with SAG1t and SA2 by combined routes. **(A,C,G)** Protection after vaccination. Protection was evaluated 1 month after challenge by analyzing the cyst load in brain tissue. Results are expressed as the mean ± SEM (*n* = 8) and represent one of two independent experiments. *P < 0.05; **P < 0.01; ***P < 0.001. Detection of specific anti-SAG1 IgG antibodies and IgG subclasses in sera of immunized mice. Serum samples were tested by ELISA using SAG1t **(B,D)**, P30 protein including the D1 domain and 17 additional amino acids **(E)** and SA1 **(F)** as the coating antigen. Results are expressed as the mean ± SEM (*n* = 8) and represent one of two independent experiments. *P < 0.05.

To determine whether combined parenteral and non-parenteral delivery routes would have a synergistic effect achieving protection against chronic toxoplasmosis, mice (8 per group) were immunized with SA1 formulated with Poly I:C by i.n., s.c. or both routes. Control mice received Poly I:C by both routes. Two weeks after the third immunization, all mice were orally infected with cysts of the 76K *T. gondii* strain. The brain cyst load was determined one month after this challenge. Compared to the control group (3,098 ± 749 cysts), parasite burden was significantly lower in mice immunized by i.n. route (1,645 ± 990), s.c. route (1,690 ± 812), and combined routes (1,350 ± 958), corresponding to a reduction in brain cyst load of 47, 45, and 56%, respectively (Figure 5A). There was no statistically significant difference between the three immunized groups; however, the difference between the control group and the group immunized by the combined routes was more statistically significant.

In parallel, the systemic humoral IgG response was analyzed, and the anti-SAG1 serum IgG subclasses were determined to assess the relative contribution of Th1/Th2 type cellular immunity. After the third immunization, mice vaccinated by the s.c. and the combined routes showed high level of specific anti-SAG1 IgG antibody titers. However, specific IgG anti-SAG1 antibodies were not detected in mice immunized i.n. (Figure 5B). The IgG antibody titer was significantly greater in the sera of mice immunized using combined routes. Predominant production of IgG1 and very low production of IgG2a were detected in sera of mice immunized by the s.c. route. IgG1 production suggests a predominant Th2 type response in these mice. On the contrary, mice immunized by the combined routes produced high levels of IgG2a and IgG1 (Figure 5B), suggesting a mixed Th1/Th2 response. Although the i.n. route alone did not induce a systemic humoral response, its combination with the s.c. route increased the production of IgG2a compared to the s.c. route alone, suggesting a more polarized Th1 type response. Thus, the combined immunization approach tends to enhance the protection against *T. gondii*.

#### SA2 Fusion Protein Vaccine Improves Protection Compared to SA1

Having determined that simultaneous i.n. and s.c. administration achieved the best protection against *T. gondii* infection, the combination of these two routes was used to evaluate the immunogenic potential of SA1 and SA2 targeted proteins in mice. Compared to the control group (3,158 ± 816 cysts), mice immunized with SA1 showed a reduction of 51% (1,240 ± 719) in the number of brain cysts, whereas mice immunized with SA2 showed even fewer cysts with an 80% (550 ± 251) reduction in brain cyst load (Figure 5C). As shown in Figure 5D, high levels of specific anti-SAG1 IgG antibodies were found in the sera of mice immunized with SA1 or SA2. However, the IgG antibody titers were significantly greater in the sera of mice immunized with SA2 than in mice immunized with SA1. Both groups produced IgG1- and IgG2a-specific anti-SAG1 IgG subclasses antibodies, suggesting a mixed Th1/Th2 type response. Comparable levels of IgG2a and IgG1 were produced in mice immunized with SA2, whereas the IgG1 level was slightly higher than IgG2a in mice immunized with SA1 (Figure 5D). In conclusion, the targeted D1 domain of SAG1, which contains both B and T epitopes, conferred protection. In addition, the presence of the D2 domain, which also contains T epitopes, potentiated the Th1 response and further improved protection.

Moreover, to compare level of anti-D1 domain antibody between SA1 and SA2 immunized mice, we used a recombinant commercial P30 protein, which included the D1 domain and 17 additional amino acids (P30: 45–198 amino acids and D1: 48–181 amino acids). As shown in Figure 5E, equal titers of IgG antibodies are detected in the sera of mice immunized with SA1 or SA2. The same results are obtained when SA1 is used as a coating antigen (Figure 5F).

In conclusion, both SA1 and SA2 immunizations induced the production of specific antibodies directed to the D1 domain at equal levels. SA2 immunization induced additional production of anti-D2-specific antibodies leading to a significant higher level of total IgG anti-SAG1.

#### Targeting Provide Better Reduction of Brain Parasite Load

The enhanced efficiency of DC targeting was demonstrated by the evaluation of protection after immunization of mice with targeted (SA2) and untargeted (SAG1t) proteins by combined i.n. and s.c. routes. As showed in Figure 5G, parasite burden was dramatically lower (80%) in the SA2 immunized group compared with control or SAG1t immunized mice (550 ± 250 cysts) (Figure 5G). The untargeted protein vaccine significantly reduce brain parasite load, achieving a decrease of only 33% (2,081 ± 463 cysts) compared with control mice (3,105 ± 821 cysts). Therefore, SAG1 targeting clearly enhances the immune response that protects mice against chronic infection.

### SAG1 Targeting Improves the Immune Response Induced

As SA2 clearly enhanced protection against chronic *T. gondii* infection compared with SAG1t, the associated humoral and cellular immune responses were further analyzed prior to the oral challenge.

#### Mucosal and Systemic Humoral Immune Responses

**Figure 6:**
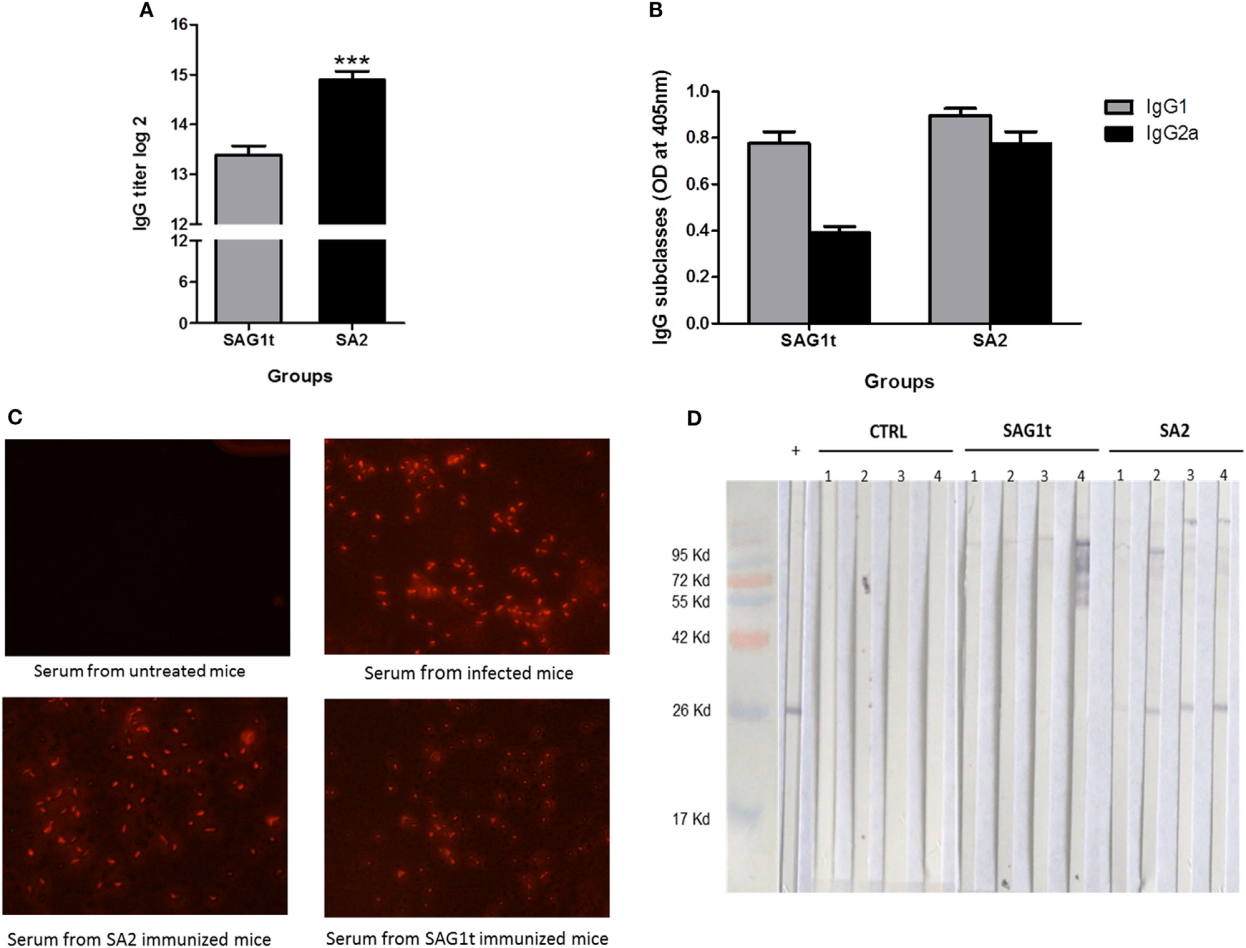
Specific antibody response in mice immunized with untargeted (SAG1t) or targeted (SA2) proteins. Detection of specific anti-SAG1 IgG antibodies **(A)** and IgG subclasses **(B)** in sera of mice (12/group) primed and boosted twice with SAG1t, SA2, or phosphate-buffered saline formulated with Poly I:C by combined routes. Serum samples collected after the last boost were tested by ELISA using SAG1t protein as the coating antigen. Results are expressed as the mean ± SEM (*n* = 12) of log2 titers and represent one of two independent experiments. ***P < 0.001. **(C)** Immunofluorescence assay of *Toxoplasma gondii* tachyzoites with sera of mice immunized with SAG1t or SA2 proteins. Tachyzoites were labeled with anti-Mouse IgG-TRITC antibody (1:200) after incubation with sera. Serum from non-infected and infected mice were used for negative and positive controls, respectively. **(D)** Western blot analysis of the IgA antibody response in nasal washes. IgA specific for SAG1 protein were analyzed 1 week after the last boost, in nasal washes from four mice of each group (control, SAG1t, and SA2).

Sera, nasal, and intestinal washes were collected to analyze the specific IgG and IgA responses, respectively. SA2 immunized mice have significantly higher IgG titer than mice immunized with SAG1t (Figure 6A). Both IgG1 and IgG2a subclasses were detected in the SA2 and SAG1t immunized groups (Figure 6B). As shown before, comparable levels of IgG2a and IgG1 were produced in mice immunized with SA2, while IgG1 level was higher than IgG2a in mice immunized with SAG1t. SAG1 targeting clearly increased the humoral immune response, especially IgG2a production. Moreover, immunofluorescence analysis showed that serum anti-SAG1 antibodies recognized tachyzoites, confirming the immunogenicity of the vaccinated proteins (Figure 6C).

Nasal and intestinal washes (four mice in each group) were analyzed by Western blot on SAG1t. Anti-SAG1 IgA antibodies were detected in the nasal washes of four mice immunized with SA2, whereas anti-SAG1 IgA antibodies were not detected in mice immunized with SAG1t nor in control mice. (Figure 6D). However, specific anti-SAG1 IgA antibodies were not detected in intestinal washes (data not shown). These data show that SAG1 targeting *via* the nasal route triggered a specific mucosal humoral response.

#### Local and Systemic Cellular Immune Responses

**Figure 7:**
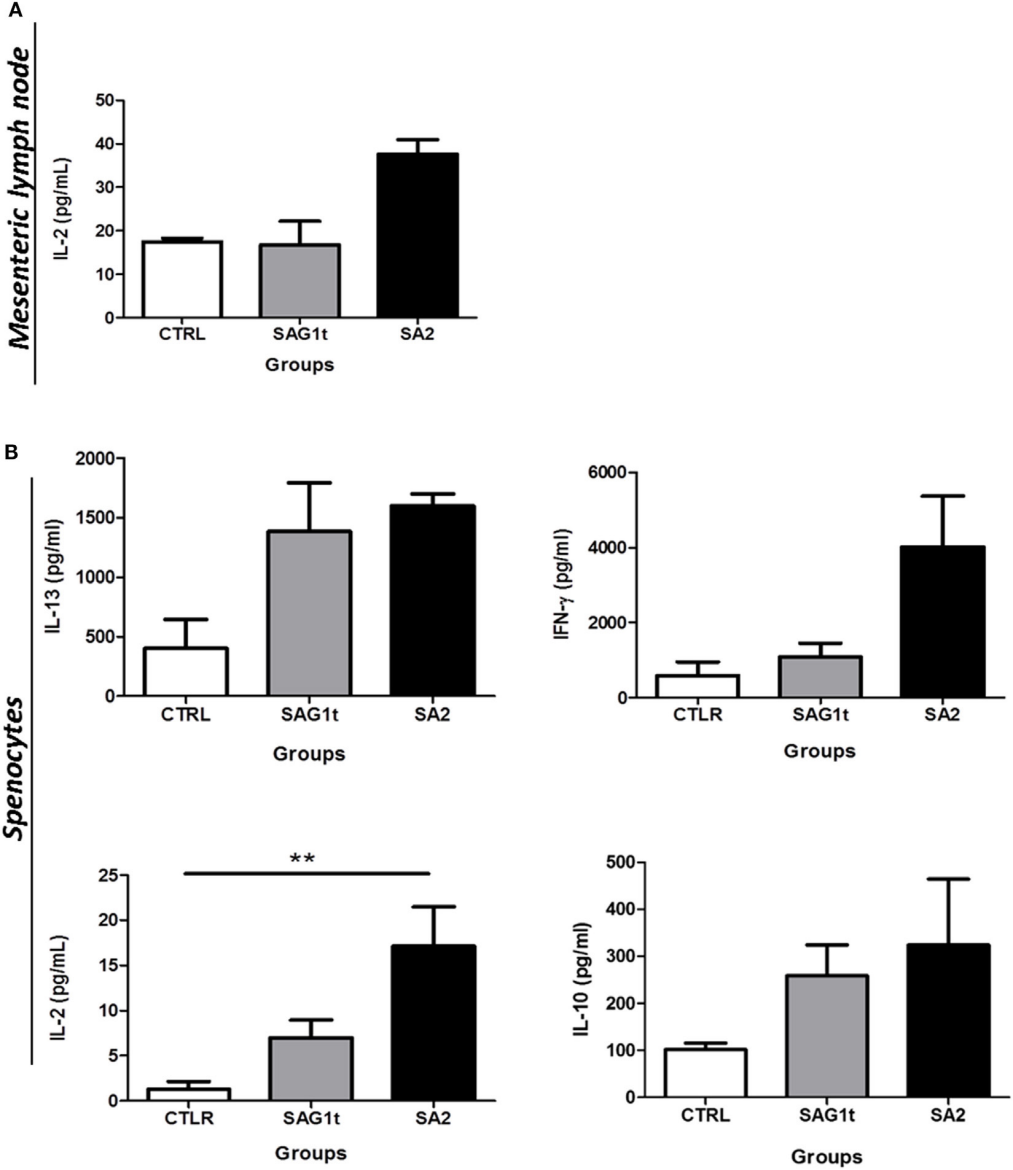
Cellular response after vaccination with SAG1t or SA2 by the combined routes. CBA/J mice were primed and boosted twice by combined intranasal and subcutaneous routes with SAG1t, SA2, or phosphate-buffered saline formulated with polyinosinique-polycytidylique acid adjuvant. 7 days after the last immunization, mesenteric lymph node cells **(A)** and splenocytes **(B)** were collected from four mice in each group and restimulated with TE. Supernatants were collected after 24 (IL-2) or 72 h (IFN-γ, IL-10, and IL-13) for cytokines assay. Results are expressed as the mean ± SEM and represent one of two independent experiments. **P < 0.01.

Splenocytes and MLN cells were stimulated with *T. gondii* extract (TE), and the levels of cytokines (IFN-γ, IL-2, IL-5, IL-10, and IL-13) were assayed in their supernatants. Only IL-2 was detected in supernatants from stimulated MLNs cells. SA2 primed cells responded to TE stimulation by higher production of IL-2 compared to SAG1t primed MLN cells; however, this production did not reach statistical significance (Figure 7A).

Stimulated splenocytes from mice immunized with SA2 and SAG1t produced IL-13 at similar levels. IL-5 was not detected. This suggests that targeting had no effect on the Th2 response. However, even if the difference did not reach statistical significance, stimulated splenocytes from mice immunized with SA2 produced more IFN-γ than those from mice immunized with SAG1t. Furthermore, stimulated splenocytes from mice immunized with SA2 produced significantly more IL-2 than those from mice immunized with SAG1t. IL-10, which controls the Th1 immune response, was produced in both groups of immunized mice at similar levels (Figure 7B). These results suggest that both SAG1t and SA2 induce a mixed Th1/Th2 immune response, and indeed, SAG1 targeting potentiated the Th1 cellular immune response.

**Figure 8:**
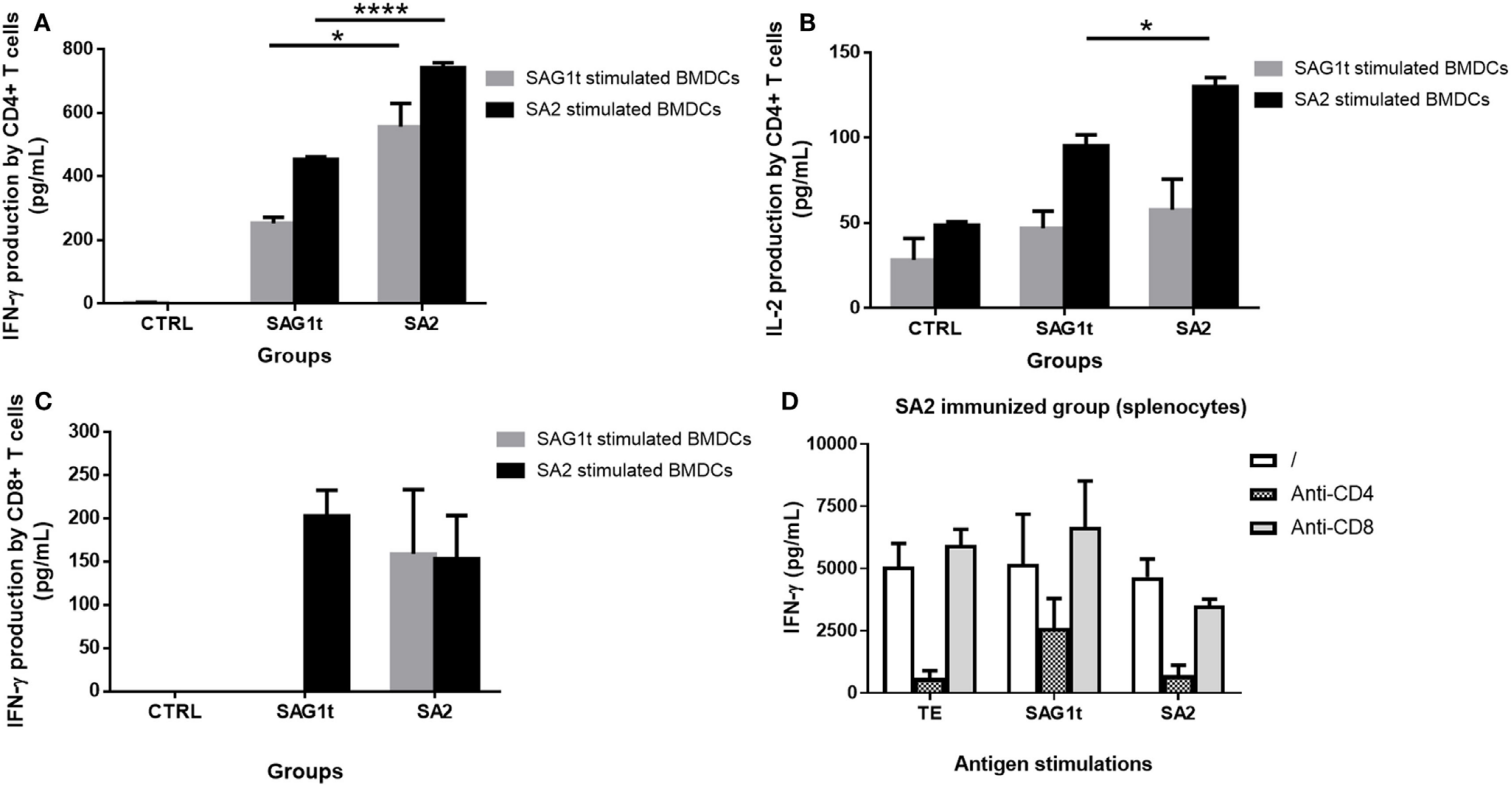
Cytokine secretion by CD4+ and CD8+ T cells from mice immunized with untargeted (SAG1t) or targeted (SA2) proteins. CD4+ and CD8+ T cells were purified from control and immunized mice and incubated with SAG1t and SA2 prestimulated bone marrow dendritic cells (BMDCs). Supernatants were collected after 72 h for cytokine assay. **(A)** IFN-γ production by CD4+ T cells, **(B)** IL-2 production by CD4+ T cells, and **(C)** IFN-γ production by CD8+ T cells. Results are expressed as the mean ± SEM. *P < 0.05, ****P < 0.0001. **(D)** Seven days after the third immunization, spleen cell suspension from SA2 immunized mice was stimulated with TE, SAG1t, or SA2 in the absence or the presence of anti-CD4 or anti-CD8 mAb. IFN-γ was measured in supernatant after 72 h. Results are expressed as the mean ± SEM.

The Th1 protective immune response was further investigated by analyzing the involvement of CD4+ and CD8+ T cells in cytokine secretions. Purified CD4+ and CD8+ T cells from control and immunized mice were cultured with BMDCs prestimulated with either SAG1t or SA2, and the production of cytokines (IFN-γ and IL-2) by the CD4+ and CD8+ T cells was analyzed. Our results showed a significant higher production of IFN-γ and IL-2 by the purified CD4+ T cells from SA2 immunized mice compared to those from SAG1t immunized mice (Figures 8A,B). Indeed, these significant higher levels were obtained when the CD4+ T cells were incubated with SA2 pulsed dendritic cells. Furthermore, in comparison to SAG1t-stimulated DCs, the SA2 pulsed DCs were able to promote the CD4+ T cell immune response of the SAG1t immunized mice. Moreover, a slight production of IFN-γ by CD8+ T cells from mice immunized with SA2 and SAG1t was also detected when BMDCs were stimulated with SA2, but there was no statistically significant difference between the two groups (Figure 8C). However, when BMDCs were pulsed with SAG1t, CD8+ T cell production of IFN-γ was only detected in CD8+ T cell supernatants from SA2 immunized mice. These results were confirmed when anti-CD4- or anti-CD8-specific mAb were added during stimulation of splenocytes from SA2 immunized mice with TE, SAG1t, or SA2. Indeed, as shown in Figure 8D, anti-CD4 mAb reduced the specific IFN-γ secretion whatever the stimulation antigen. In contrast, adding anti-CD8 mAb to the cultures reduced slightly the IFN-γ secretion only when spleen cells were stimulated with SA2.

These results suggest that SAG1 targeting to DEC205 dendritic cells receptor resulted in efficient MHC class II and I restricted antigen presentation and improved mainly a CD4+ T cell immune response and to a lower extend a CD8+ T cell immune response.

## Discussion

Antigen targeting to DCs is an elegant approach to improve vaccine efficiency that has been intensively investigated in the past 10 years. For the first time, we show that SAG1 (*T. gondii* antigen) targeting to DEC205+ dendritic cells v*ia* a scFv fragment by i.n. and s.c. administration improved the protection against chronic *T. gondii* infection. A strongly reduced brain parasite burden was observed when compared to the i.n. or the s.c. route alone. DC targeting improved both local and systemic humoral and cellular immune responses and potentiated the Th1 response profile in particular, through more efficient production of IFN-γ, IL-2, IgG2a, and nasal IgA.

If s.c. immunization triggered systemic immune response, i.n. route improved both systemic and mucosal immune responses (14, 15, 41). Mucosal immunity is the first line of defense against *T. gondii*, which naturally invades the intestine of its host. Systemic immunity is important in protecting against parasite dissemination especially in the case of fetal transmission or parasite reactivation. Under our conditions, systemic or i.n. routes alone protected mice from chronic toxoplasmosis, but the protection was more effective when the routes were combined. The combination of the two routes, eliciting both systemic and mucosal immune responses, was explored by intradermal and sublingual vaccinations (34) and more recently by i.n. and intramuscular (35) or i.n. and intradermal routes (36). In this last study, mice primed intradermally with serine protease inhibitor 1 (TgPI-1) plus alum and boosted i.n. with TgPI-1 plus CpG-ODN showed a 62% reduction in the brain parasite burden after oral challenge with cysts of the ME49 *T. gondii* strain. A strong Th1/Th2 protective systemic response was induced along with a mucosal immune response characterized by specific intestinal IgA.

The protective efficiency against toxoplasmosis depends on the Th1 immune response (42). Adoptive transfer of T cells from the spleen (43) and experiments with mice depleted of T cells (44) indicate that CD4+ and CD8+ T lymphocytes help mediate resistance to *T. gondii*, probably through IFN-γ production. More specifically, the ability of the induced anti-SAG1 immune response to protect naive recipient mice against toxoplasmosis was demonstrated by cell transfer experiments using cervical (CLN) and MLN cells (45). These experiments showed that CLN and MLN cells transferred protective immunity from SAG1 i.n. immunized mice to naive mice, significantly reducing the cyst load (60%) after challenge. Moreover, adoptive transfer of CD8+ T cells from mice immunized with a plasmid encoding SAG1 to naive mice showed partial protection (46).

SAG1 targeting to DEC205 dendritic cell receptor resulted in efficient MHC class II restricted antigen presentation and improved mainly a CD4+ T cell immune response, specifically the Th1 response profile by more efficient production of IFN-γ and IL-2. As mouse CD8α+ DCs are superior at cross-presentation, targeting this DC subset could theorically be an efficient way to induce CD8+ T cell immune responses. The *in vitro* analysis of antigen presentation by SAG1t- and SA2-stimulated dendritic cells showed that DEC205 targeting improved MHC I SAG1 antigen presentation, both CD8+ T cells from SAG1t and SA2 immunized mice produced IFN-γ when incubated with SA2-stimulated dendritic cells, while only CD8+ T cells from mice immunized with SA2 produced IFN-γ when incubated with SAG1t stimulated dendritic cells. Consequently, whatever the DCs stimulation antigens, mice immunization with targeted SAG1t improved the CD8+ T cell immune response. However, the CD8+ T cell immune response of SAG1t immunized mice was detectable only following incubation with SA2-stimulated dendritic cells.

Further, 7 days after one immunization, *in vivo* analysis of the T lymphocyte subsets by flow cytometry showed a slight increase in the percentages of CD4+ T cells in MLN and inguinal lymph nodes from SA2 immunized mice (62 and 53%, respectively) compared to SAG1t immunized mice (55 and 47%, respectively). No change in the percentages of CD8+ T cell was found in MLN and inguinal lymph nodes. Similar percentages of CD4+ T cell were found in spleen from SA2 and SAG1t immunized mice. After three immunizations, the number of activated CD4+/CD69+ and CD8+/CD69+ T cells in the spleens was slightly higher in mice immunized with SA2 (5.7 and 3.8%, respectively) relative to those immunized with SAG1t (4.7 and 2.8%, respectively) (data not shown). Thus, SAG1 targeting potentiated the CD4+ T cell immune response at the local level after one immunization and promotes both of CD4+ and CD8+ T cell response at the systemic level following three immunizations.

These results are in agreement with those obtained by others showing that in combination with TLR stimulation, DEC205 targeting induced a robust CD4+ T cell immune response (47–49) and only low level of CD8+ T cell response (25, 40). One exception is OVA protein for which both potent CD4+ and CD8+ responses were obtained (39, 50, 51). In fact, previous reports have more focused on mapping CD4+ T cell epitopes to SAG1t. Prediction studies have identified some MHC I restricted epitopes (H-2^k^, H-2^b^ haplotypes) in the SAG1 sequence (46, 52). Four peptides (H-2^k^ haplotype) were used to detect specific CD8+ T cells secreting IFN-γ following immunization of mice (C3H) with a plasmid DNA encoding SAG1 and only one showed a specific response in an ELISPOT assay. The low CD8+ T cell response can be explained by the low frequency of T CD8+ epitopes (H-2^k^ haplotype) on SAG1t sequence and/or the low number of antigen-specific CD8+ T cells generated in our experimental conditions.

Under our conditions, mice immunization with SAG1 protein induced a strong humoral response and a moderate cellular response, which only gave partial protection (32% reduction in brain cysts). This response was improved with antigen targeting, which polarized the induced immunity toward a Th1 profile, increasing IFN-γ and IL-2 production. IFN-γ controls both acute and chronic infection, restricts the growth of parasites in the acute phase, and prevents reactivation of parasites from dormant cysts at a later phase. The IL-2 cytokine can increase IFN-γ production, and both cytokines can modulate IgA production (52). Under our experimental conditions, the production of IFN-γ and IL-2 in SA2 vaccinated mice correlated with IgA production.

It has also been shown that antibody titers are much increased by the greater CD4+ T cell activation obtained with DEC205 targeted antigen (53, 54). DCs play an important role in B cell activation because they are at the origin of differentiation of T helper lymphocytes, which provide complementary signals to B lymphocytes *via* CD40L and IL-4. The possibility that DCs are directly involved in B cell activation has been proposed previously through the presentation of opsonized antigens or the regurgitation of native antigen from their intracellular compartments (55). It has also been reported that monoclonal anti-SAG1 antibodies can inhibit infection of host cells *in vitro* and that opsonized-tachyzoites are more easily neutralized by macrophages (12). In this study, the obtained protection was correlated with high titers of anti-SAG1 systemic IgG.

The domain D1 of SAG1 contains several B (7, 55) and T epitopes (8, 55). Furthermore, an anti-SAG1 neutralizing antibody, recognized the D1 domain (data not shown), and this suggests that D1 domain immunization may induce neutralizing antibodies. So, it was interesting to evaluate the vaccine potential targeting of D1 domain, and obviously, our results showed that D1 targeting protected mice and significantly reduced 60% of the brain parasite burden. Targeting the D2 domain further improved the protection (80% brain cyst reduction), possibly due to the presence of T epitopes in this domain (37). Both SA1 and SA2 immunizations induced the production of specific antibodies directed against B epitopes of the D1 domain at equal level, suggesting that the difference in antibody titers between SA1 immunized mice and SA2 immunized mice is due to a specific response directed to the D2 domain. Indeed, antigenic B epitopes are distributed along the entire SAG1 sequence (56). Furthermore, we previously identified a conformational and neutralizing epitope in the central part of SAG1, located mainly on the D2 domain (57). Remarkably, the D2 domain not only improved protection but also stabilized the fusion protein. Indeed, both SA1 and the scFv fragment aggregated at a concentration of 150 µg/mL (3.7 µM), and the yield of production of SA1 was only (8 mg/L) compared to SA2 where a concentration of 17 mg/mL could be obtained.

DC targeting through the DEC205 receptor *via* the i.n. route has also been effective in improving mucosal immunity against pneumonic plague (58). Moreover, by using the i.n. route, Bonenfant et al. (15) showed that CBA/J mice immunized with SAG1, in association with cholera toxin as a mucosal adjuvant, significantly reduced the development of cerebral cysts (about 80% of brain parasite burden reduction). Unfortunately, cholera toxin is unusable, despite being a potent mucosal adjuvant. Interestingly, under our experimental conditions, DC targeting was as effective as i.n. administration of SAG1 plus cholera toxin in promoting protection. The i.n. immunization with SAG1 and cholera toxin induced IgA expression in the nasal and intestinal mucosa. In our study, IgA was only detected in nasal washes.

Induced mucosal immunity can be optimized. Increasing the immunization dose has been investigated but did not improve protection (data not shown). Another possible strategy is the use of a simultaneous nasal adjuvant delivery system. Dimier-Poisson et al. (33) developed DGNP nanoparticles able to deliver proteins within airway epithelial cells. Nanoparticle/*T. gondii* antigens formulation by nasal vaccination elicits high protection against toxoplasmosis regarding survival and parasite burden, correlated with an increased delivery of antigens by DGNP nanoparticles in airway mucosa cells. Moreover, glucopyranosyl lipid A (GLA), a new synthetic non-toxic analog of lipopolysaccharide induced higher antibody titers and generated Th1 cell responses when formulated with HIV targeted antigen to DEC205 and administered s.c. (11). GLA elicited also protective immune response when administered with *T. gondii* GRA7 epitopes (23). Therefore, the formulation of targeted SAG1 with GLA and/or nanoparticles can improve protective immunity against toxoplasmosis.

It has been demonstrated that the induction of CD8+ T cell response against *T. gondii* depends on the antigen location and that secreted proteins are the potential inducers of CD8+ T cell response (59). On one side, targeting secreted proteins as GRA, AMA1, and ROP may enhance protective immunity against *T. gondii*. Thus, the targeting of an antigenic *T. gondii* complex could probably improve the protection. Antigenic complex vaccination has been effective against toxoplasmosis with gene vaccination (38, 60). On the other side, targeting other endocytic receptors such as CLEC9A or XCR1 could be relevant to target more specifically BDAC3 human/CD8α+ dendritic cells. Targeting several populations of DCs has also been investigated recently and probably it could be a good promising strategy (61).

Summarizing, i.n., and s.c. immunizations of CBA/J mice with SAG1 targeted protein administered with TLR3 agonist provided strong protection against chronic *Toxoplasma* infection. For the first time, this study demonstrates the potential of a DC-targeted vaccine as a novel approach against chronic toxoplasmosis infection, and this can be applied to other *Apicomplexa* parasites.

## Ethics Statement

The animals were maintained under standard conventional conditions and all experimental procedures were conducted to the guidelines for the animal experimentation and the protocol was approved by the local ethics committee (CEEA VdL).

## Author Contributions

NA, M-NM, ZL, and ID-P conceived the study design. ZL performed the experiments, analyzed the data, and wrote the manuscript. NA, M-NM, and ID-P contributed at data analysis and the manuscript writhing. M-NM and AM helped in the implementation of the study. BH performed the animal experiments. NM contributed to cytometry data analysis and the final review of the article. AD-T and MJ contributed to the final review of the manuscript. All authors read and approved the final manuscript.

## Conflict of Interest Statement

The authors declare that the research was conducted in the absence of any commercial or financial relationships that could be construed as a potential conflict of interest.
